# Making the cut: The production of ‘self-harm’ in post-1945 Anglo-Saxon psychiatry

**DOI:** 10.1177/0952695112473619

**Published:** 2013-04

**Authors:** Chris Millard

**Affiliations:** Queen Mary, University of London, UK

**Keywords:** disease concepts, gender, history, psychiatric syndromes, self-harm, self-mutilation, stereotypes

## Abstract

‘Deliberate self-harm’, ‘self-mutilation’ and ‘self-injury’ are just some of the terms used to describe one of the most prominent issues in British mental health policy in recent years. This article demonstrates that contemporary literature on ‘self-harm’ produces this phenomenon (to varying extents) around two key characteristics. First, this behaviour is predominantly performed by those identified as female. Second, this behaviour primarily involves cutting the skin. These constitutive characteristics are traced back to a corpus of literature produced in the 1960s and 1970s in North American psychiatric inpatient institutions; analysis shows how pre-1960 works were substantially different. Finally, these gendered and behavioural assertions are shown to be the result of historically specific processes of exclusion and emphasis.

## Introduction

A number of mental health concerns during the 1990s crystallized around ‘Prozac’, cohering and refracting heterogeneous issues around ‘depression’, psychoactive medication and the influence of pharmaceutical companies (Wurtzel, 1994; Healy, 1997; Shorter, 1997). In a significant shift, the first decade of the 21st century might be characterized as the ‘decade of self-harm’. Mental health workers have been among the most prominent producers of an avalanche of information attempting to categorize, analyse, explain and treat ‘self-harm’. Since 2004 the Royal College of Psychiatrists has produced no fewer than four reports on the issue (Royal College of Psychiatrists, 2004, 2006, 2008, 2010). A set of National Health Service guidelines on the treatment of self-harm jointly produced by the National Institute for Clinical Excellence (NICE) and the National Collaborating Centre for Mental Health (NCCMH) sparked a media furore in 2004, as did a 2006 debate at the Royal College of Nursing Congress, about ‘the nurse’s role in enabling patients to self-harm safely’.^1^

More broadly, the pop music sub-genre ‘emo’ became associated with self-harm (as had ‘Nu-Metal’ before it). The* Daily Mail* carried an article in August 2006 entitled ‘EMO Cult Warning for Parents’, arguing that ‘emo’ is a ‘dangerous teenage cult’ and that one of its key features ‘is a celebration of self harm’ (Sands, 2006). Actor and writer Meera Syal presented a documentary on ‘self-harm’ entitled ‘A World of Pain’ shown on BBC2 in June 2009, and recent book-length studies include Adler and Adler’s *The Tender Cut* (2011), Tantam and Huband’s *Understanding Repeated Self-Injury* (2009) and the 3rd edition of Jan Sutton’s *Healing the Hurt Within* (2007). A cursory search of internet news stories limited to September 2011 revealed ‘self-harm’ stories from Fox News to the *Yeovil Express* (Fox News, 2011; *Yeovil Express*, 2011). This is not new in any simple sense, however, as is aptly demonstrated by the iconic early-1990s image of Manic Street Preachers lyricist Richey Edwards with the slogan ‘4 REAL’ carved into his forearm, or the 1970s punks that Dick Hebdige notes exhibited ‘a penchant for self-laceration’ (Hebdige, 1979: 25).

This article makes two interrelated points. First, it demonstrates that the contemporary phenomenon of ‘self-harm’ is rooted in, and substantially created through, a relatively discrete corpus of studies issuing from North American psychiatric inpatient facilities in the late 1960s and early 1970s. Second, it shows that these 1960s–1970s articles expend considerable intellectual and practical effort to establish a stable ‘syndrome’. This effort foregrounds young, feminine patients along with acts of ‘cutting’; it excludes or significantly subordinates other symptoms or patients. This article offers an explanation for these subordinations and exclusions. The current phenomenon of ‘self-harm’ is thus shown to be a relatively recent invention, and a highly specific historical object, despite the claims made in recent literature for its transcendental, ever-present status. The article also contains an implicit feminist thread throughout. This syndrome unequally pathologizes those identified as female; unpicking its gendered construction is a contribution towards exposing this inequality. More broadly, this is an attempt to write history as ‘critique’, to ‘open the possibility for thinking (and so acting) differently’ (Scott, 2007: 23).

## Current literature: ‘Self-harm’ as ‘female cutting’

This phenomenon of ‘self-harm’, ‘repeated self-injury’, or ‘self-mutilation’ appears reasonably stable in professional and popular registers; there are a number of characteristics that recur predictably under these different headings. The relative stability of the descriptive terms fosters confidence that broadly ‘the same thing’ is being described. While there are debates on whether ‘self-poisoning’ (or ‘overdosing’) should be included, or whether a certain level of ‘suicidal intent’ disqualifies behaviour from being ‘self-harm’ (Tantam and Huband, 2009: 1, 12; Sutton, 2007: 105–14), these debates are very much peripheral to the central project of psychological treatment, epidemiological analysis and (more recently in Britain) changing staff attitudes at accident and emergency departments.

This stability is interrogated historically here, traced back to a point, or set of points, in the 1960s, from where the modern psycho-clinical object of ‘self-harm’ originates. The stability is addressed through analysis of two structural characteristics^2^ that run through this phenomenon, making it distinctive, separate and stable – making it an object for analysis. These two characteristics are that ‘self-harm’ is an activity carried out principally by those gendered female, and that the stereotyped behaviour involves cutting the skin. There is nothing particularly special about these characteristics – they are necessary for any class of objects to exist. They are the particular emphases that differentiate tables from chairs, mammals from reptiles, or depression from schizophrenia: they *structure* – produce as comprehensible – the phenomena that they purport to *describe*.

To this end Adrian Wilson reminds us that ‘concepts-of-disease, like all concepts, are human and social products which have changed and developed historically, and which thus form the proper business of the historian’ (Wilson, 2000: 273). Ludmilla Jordanova observes that this theoretical position opens up myriad intellectual and analytical possibilities: ‘[b]y stressing the ways in which scientific and medical ideas and practices are shaped in a given context, it enjoins historians to conceptualise, explain and interpret the processes through which this happens’ (Jordanova, 2004: 340).^3^ The characteristics that structure ‘self-harm’ have not always been what they are now – they have a history.

The *Independent on Sunday* (2009) warns that it is specifically *young women* who are self-harming with alarming frequency: ‘the number of people harming themselves deliberately has leapt by a third in the past five years … the biggest rise in self-harm and attempted suicide has been among young women between the ages of 16 and 24’. (The newspaper associates these figures with World Health Organization [WHO] research on mental health and inequality.) *Yeovil Express* (2011) reports almost identical findings from the report ‘Suicide and Self-harm in the South West’. The Royal College of Psychiatrists reports that ‘[o]f those who present at hospitals, two-thirds of patients who self-harm are under the age of thirty-five years and two-thirds of people in this age group are female. Overall, women are more likely to self-harm than men’ (Royal College of Psychiatrists, 2010: 22). Jan Sutton comments that ‘[i]n the main, research suggests that self-injury is more common among females’ (2007: 41). Feminization is achieved in Marilee Strong’s *Bright Red Scream* through her stated policy of using ‘the feminine pronoun in all third person references to those who cut, since the vast majority of cutters are female’ (2000: viii). There are also bald assertions that discount any perceived variation, as in Lori Plante’s *Bleeding to Ease the Pain*: ‘females self-injure at a higher rate than males’ (2007: 15–16). Additionally, all the people featured in Meera Syal’s documentary are women (although one man is mentioned in passing).

The existence of the gendered stereotype is most clearly demonstrated by attempts to address it directly; for example: ‘Self-harmers “include boys too”’ (British Broadcasting Corporation News, 2008). Jan Sutton attempts to explode the ‘myth’ that it is only a ‘female behaviour’. She claims that ‘it is important not to lose sight of the fact that males do self-injure, despite them appearing to be in the minority’. However, she undermines this position, and her subsequent analysis goes on to reinforce existing stereotypes: ‘Why the possible genders divide? … Common theories about why men are in the minority centres on differences in socialisation.’ From ‘appearing to be’ and ‘possible’, we end up with ‘men *are* in the minority’ (Sutton, 2007: 41; emphasis added). There is some awareness that rather crude stereotyping pervades these presentations of ‘self-mutilation’. Liz Frost notes in *Young Women and the Body* that the ‘early research trends, which suggest that these problems are just the province of pretty, clever, white, middle-class girls, are being seriously challenged for their stereotyping’. She rightly castigates the ‘tendency to ignore issues of class, sexuality and colour’, but omits gender (Frost, 2000: 22). Plante qualifies the femininity of ‘self-mutilation’ rather apologetically: ‘[n]onetheless, young men do self-injure, do suffer from eating disorders, and do experience the same emotional turmoil and perceived inadequacy more commonly exhibited in women’ (2007: 16).^4^

Digby Tantam and Nick Huband are perhaps the most equivocal in their appraisal of the gendering of this behaviour, finding that ‘community studies are inconsistent about whether self-injury is more common in boys than girls’ (2009: 4).^5^ However, their work opens with one of the clearest statements of differentiation between ‘self-injury’ and ‘self-poisoning’ – they disqualify themselves from commentary on the latter – which gives the object of their research similar stability:

This book focuses on people who repeatedly injure themselves by cutting, burning, or otherwise damaging their skin and its underlying tissue. This ‘self-injury’ is one of the two main types of self-harm, the other being self-poisoning with household or agricultural chemicals, or with medication. … Self-injury and self-poisoning are often regarded as sufficiently similar to be considered as two facets of one problem. This fits with the observation that many of those who cut themselves also take overdoses, but it is not consistent with the very different cultural and psychological roots of self-injury and of self-poisoning. (Tantam and Huband, 2009: 4)

As well as the explicit differentiation, they compress the varied ‘*cutting*, *burning*, or *otherwise damaging* their skin and its underlying tissue’ into ‘many of those who *cut* themselves’ (Tantam and Huband, 2009: 4, emphases added); their chosen object of intervention – as they participate in its (re)production – is narrowed further. This is done explicitly by Jan Sutton’s question-and-answer around the term ‘*self-inflicted injuries*’. This is a term used generically (and in her view, misleadingly) in the media to talk about statistics from studies that include both ‘cutting’ and ‘overdosing’: ‘What sort of image does that [term] conjure up? Overdosing? I doubt it. Cutting? Highly probable.’ She explicitly closes ‘self-injury’ down into one specific behaviour with supreme confidence: ‘mention the word “self-harm” and it immediately conjures up images of people cutting themselves’ (Sutton, 2007: 105–6). The proposed revisions to the *Diagnostic and Statistical Manual of Mental Disorders* (*DSM-5*) include an independent ‘self-harm’ category for the first time; ‘Non-Suicidal Self-Injury’ is described as ‘intentional self-inflicted damage to *the surface of his or her body*’, ruling out ‘overdosing’, for example (*Diagnostic and Statistical Manual of Mental Disorders:* *Proposed Revisions*, 2011).

However, Liz Frost reminds us that ‘[t]his disputed term – self-harm, deliberate self-harm and self-mutilation, being the three most commonly used – can refer to a variety of behaviours’ (2000: 19). Jane Hyman, author of *Women* *Living with Self-Injury*, concurs: ‘Self-injury is as complex and diverse as the women who experience it and it seems to defy any single descriptive term’ (1999: 9). Nevertheless, continued emphasis on one particular behaviour is pervasive.

Louis Arnold, author of a number of pamphlets on ‘self-injury’ for the Bristol Crisis Centre for Women, is explicit: ‘The most common [form of ‘self-injury’] is probably cutting, often of the arms, as well as many other areas. Cuts are usually quite superficial’ (Arnold, 2002: 2). Armando Favazza and Karen Conterio state of their ‘typical cutter’: ‘Skin cutting is her usual practice’ (1989: 283). The Royal College of Psychiatrists claims that ‘the most common form of self-injury is cutting’ (2010: 21) and the explicitly psychoanalytic Fiona Gardner claims in *Self Harm: A Psychotherapeutic Approach* that ‘[i]t is well-recognized that self-harm, particularly cutting, is prevalent in some crowded institutional settings’ (2001: 137). Additionally, Syal’s documentary features a bloody razor-blade in the title shot, demonstrating the collapse of ‘self-harm’ into ‘cutting’. More slippages occur from general ‘injury’ to specific ‘cutting’, as Plante does even as she starts to problematize the gendered aspects of this phenomenon:

Men’s unwillingness to exhibit *self-injury* may result in under-reporting … [a]s one young male *cutter* said during a treatment session, ‘My friends would think cutting is really gay.’ (Plante, 2007: 16; emphases added)

She is also explicit about collapsing ‘self-injury’ into one possible type of the behaviour: ‘[c]utting, often referred to interchangeably in this book as *self-harm *or *self-injury*’, and again: ‘self-injury, also referred to interchangeably as *cutting *and *self-mutilation*’ (Plante, 2007: xiii; original emphases plus one emphasis added).

This object for psychiatric, psychological and sociological intervention draws its stability, its distinctiveness, *its very existence* from these two characteristics. ‘Self-harm’ in the early 21st century is principally about ‘young women’ cutting themselves.

## ‘Female cutting’ from an historically specific corpus of studies

Many of these texts do not treat the behaviour historically (e.g. Hyman, 1999; Sutton, 2007; Tantam and Huband, 2009). Those that do – principally academic psychology and sociology – draw unsustainable, transcendental linkages across centuries, while deploying almost identical narratives from 1800 onwards. Plante argues that ‘[c]utting is not simply a bizarre new phenomenon of the twenty-first century. … Throughout ancient and modern history and across primitive and contemporary cultures, self-inflicted bodily damage has been an important and highly symbolic act. … History abounds with innumerable examples’ (2007: 5–6). Adler and Adler open their book with the concise statement that ‘Self-injury has existed for nearly all of recorded history’ (2011: 1). This conforms to Adrian Wilson’s description of the dominant approach to the history of medicine, where

… diseases *throughout history *have been assigned their *modern* names-and-concepts … *responses *to diseases are allowed to vary historically; but this historiographic permission is withheld from the diseases themselves … modern disease concepts … have been assigned a transhistorical validity. (Wilson, 2000: 273)

The most epic and indulgent example of this brings together the Passion of Jesus Christ, Tibetan tantric meditation, North American Plains Indian mysticism and the writings of Franz Kafka and the Marquis de Sade (among others) in a ‘history’ of self-mutilation (Favazza, 1996: 2, 11–16). In accounts such as this, in Wilson’s words, ‘the historicity of all disease concepts, whether past or present, has been obliterated’ (2000: 273).

This is a dizzying array of literature, covering academic sociology, self-help, internet ethnography, popularizing texts, and books for practitioners, counsellors and clinicians. In all of the writing on this supposedly ‘hidden’ subject, there appears only one sensitive historical treatment, critiquing the unchanging profile. Barbara Brickman argues that ‘[i]n the late 1960s and 1970s a cutter profile was created … typically a white, adolescent girl’. She points out that ‘that picture of the typical “cutter” appears again and again in popular articles and fiction’ (Brickman, 2005: 87). As we have seen, we can add psychiatric, sociological and self-help texts to the popular registers with which Brickman is concerned. However, for all her awareness of gender concerns, she reproduces the collapse of ‘varied mutilations’ into ‘cutting’:

… [e]xcluding other forms of self-mutilation such as burning, head-banging, self-biting, ingestion of harmful items and chemicals, etc. I selected delicate self-cutting because it is one of the most frequently observed (and reported) forms of self-mutilation and because of the instances of cutting in popular media. (Brickman, 2005: 90)

She therefore discounts the idea that the behavioural rather than the gendered stereotype is worthy of investigation. She too gives ‘cutting’ pride of place.

The connection between the late 1960s and the present is more extensive and systematic than Brickman demonstrates. Modern texts’ references lead to a coherent corpus of studies from the late 1960s and 1970s, issuing principally from a set of psychiatric institutions in the north-eastern USA. A comprehensive survey in 1976 mentions ‘classical studies’ of self-mutilation published in the late 1960s (Simpson, 1976: 290).^6^ These are all studies published in academic clinical journals, aimed at physicians and psychiatrists. There is very little literature on self-harm outside of this rather rarefied space during the 1960s, apart from a couple of references to Hannah Green’s (Joanne Greenberg’s) novel *I Never Promised You a Rose Garden* (1964) (in Graff, 1967: 62 and Rinzler and Shapiro, 1968: 487) and Sylvia Plath’s 1962 poem ‘Cut’ (in Simpson, 1976: 304 and quoted on the front page of Waldenburg, 1972).

These ‘classical studies’ refer to each other to a substantial extent (especially Pao, 1969; Burnham, 1969; Nelson and Grunebaum, 1971; Asch, 1971; Rosenthal, Rinzler, Wallsch *et al.,* 1972), and these texts became increasingly aware of each other. This coherence was no doubt aided by the fact that 13 of these studies issued from just four institutions: the Eastern Pennsylvania Psychiatric Institute, Massachusetts Mental Health Centre, Mount Sinai Hospital (New York) and Chestnut Lodge (Maryland). This coherence and awareness built up the sense of significance and discreteness implied by Simpson’s description of them as ‘classical’. However, this author initially discovered them independently of Simpson’s literature review, combing the references of contemporary texts (e.g. Walsh and Rosen, 1988; Cross, 1993; Favazza, 1996; Strong, 2000; Gardner, 2001; Plante, 2007) and then using the references of the 1960s–1970s studies to find more. This led back to five studies, all published in 1967 (Crabtree, 1967; Goldwyn, Cahill and Grunebaum, 1967; Graff and Mallin, 1967; Graff, 1967; Grunebaum and Klerman, 1967). The texts that constitute this corpus are regularly – and even reverently – cited in the contemporary literature up until 2007, after which point they seem to fall away somewhat, a point addressed below.

The main studies are briefly sketched out here and their links to the contemporary literature demonstrated. Harold Graff, a psychiatrist with varied interests including addiction, recreational drugs and issues of social class in psychoanalysis, wrote a key paper, ‘The Syndrome of the Wrist Cutter’, in 1967 with his colleague Richard Mallin (Graff and Mallin, 1967). In the same year, Harvard psychiatrists Henry Grunebaum and Gerald Klerman produced the article ‘Wrist Slashing’ (Grunebaum and Klerman, 1967). Both of these texts are prominently quoted in Walsh and Rosen’s *Self-Mutilation* which adds that ‘[s]upport for the idea of a separate syndrome of wrist cutting (found among young, single women with a consistent psychological and historical profile) appears to have been considerable through the late 1960s and early 1970s’ (1989: 22–3). Marilee Strong asserts that Graff and Mallin’s work is ‘one of the best early studies on cutters’ and ‘groundbreaking’, and that this, along with Grunebaum and Klerman’s article, represents ‘stunning findings [which] sparked an interest’ (2000: 32–3, 59). Plante also references these two texts when discussing developments in the 1960s (2007: 7–8).^7^ The fact that the ‘wrist’ part of the archetype seems to have disappeared does not trouble anybody (for analysis of this, see Millard, 2007).

Another significant development in 1967 was a symposium entitled ‘Impulsive Self Mutilation’ (Pao, 1969; Kafka, 1969; Podvoll, 1969; Burnham, 1969) held at Chestnut Lodge, ‘a small, private psychiatric hospital in Rockville, [Maryland] specializing in the long-term residential treatment of severely ill (and usually chronic) psychotic and borderline patients’ (McGlashan, 1984: 573). Despite its size, Chestnut Lodge was a highly influential psychoanalytic institution, ranking alongside the Menninger Clinic in Kansas. Not only was it the setting for the above-referenced series of studies on the outcomes of various psychiatric conditions (the ‘Chestnut Lodge Studies’) by Thomas McGlashan, it was the site for Alfred Stanton and Morris Schwartz’s *The Mental Hospital* (1954), a study of ‘institutional participation in psychiatric illness’, which was ‘the first of a series of monographs that were now published on the subject’ (Scull, 2011: 75–6). (Green[berg]’s *I Never Promised You a Rose Garden* (1964) semi-autobiographically recounts her experiences at Chestnut Lodge under prominent analyst Frieda Fromm Reichmann.)

The three main participants in the 1967 symposium were Ping-Nie Pao, a specialist in the treatment of schizophrenics, John S. Kafka, whose interests lay in the experience of time and reality in mentally ill patients, and Edward Podvoll who, among other achievements, founded the Windhorse Project, an experimental psychiatric community. Robert C. Burnham (described upon his death by Kafka as a ‘psychoanalyst’s psychoanalyst … a superb teacher and a subtle clinician’ [Washington Psychiatric Society, 2008]) chaired the subsequent discussion. Fiona Gardner calls the Chestnut Lodge symposium ‘[o]ne of the most valuable collections of papers’ (2001: 21). Lori Plante uses Pao’s term ‘delicate self-cutting’ without referencing him (2007: 7); Gardner takes this loaded word from Pao explicitly (Gardner, 2001: 7). A BBC article buries this term in a list without comment: ‘[t]he practice of self-harming is known by other names – self-inflicted violence, self-injury, delicate cutting, self-abuse or self-mutilation’ (Erlam, 2010).

In Favazza’s study with Karen Conterio, it is claimed that ‘most of the detailed information about chronic self-mutilators has come from a handful of psychoanalytically oriented case studies’ (1988: 23) including a case study by Loren Crabtree Jr (1967), who later worked on the psychoanalytic concept of ‘transference’ with Harold Graff in Pennsylvania (Graff and Crabtree, 1972). Favazza and Conterio’s list of references also contains a predominantly theoretical article by Peter Novotny (1972) who worked at the famous Menninger Clinic in Kansas; J. S. Kafka of the Chestnut Lodge symposium is also named (Favazza and Conterio, 1988: 23). Lori Plante acknowledges a change in the 1960s, arguing ‘modern psychiatric attention to self-injurious patterns of cutting and burning only emerged in the 1960s’ (2007: 8). Adler and Adler state broadly that ‘[s]tudies from the 1960s to the 1980s then noted the rise of “wrist-cutting syndrome,” associating it with unmarried, attractive, intelligent young women’ (2011: 14; they reference Graff and Mallin, 1967; Grunebaum and Klerman, 1967; Pao, 1969; Asch, 1971; Rosenthal, Rinzler, Wallsch *et al.,* 1972). In other words, this relatively discrete and self-referential corpus of psychiatric texts produced ‘self-injurious patterns’ in new and distinctive ways. Many of the contemporary texts are rooted in this corpus, *explicitly* tracing its concerns back to these 1960s–1970s studies.

The year 1967 seems to have been an important one, with the Chestnut Lodge symposium being held (though published in 1969), and the much-referenced papers by Grunebaum and Klerman and by Graff and Mallin being published. In addition, an article on the subject by Goldwyn, Cahill and Grunebaum, Crabtree’s case study, and another offering from Graff, entitled ‘The Chronic Wrist Slasher’ (1967), made it into print. These texts produced a very specific rendering of self-mutilation.

## The specificity of 1960s ‘self-harm’: ‘An attractive young woman’ and the ‘primary symptom of the slash’

Despite the above-mentioned claims for the virtual omnipresence of this behaviour this article now demonstrates just how much the current ‘self-harm’ archetypes are a product of the 1960s. It is not the case, of course, that discussions on ‘self-mutilation’ started in the 1960s, in a vacuum. The subject has broad ancestry at least as far back as the 19th century (for example, Chaney, 2011a, 2011b). Indeed, many of the recent sociological and self-help texts refer to American psychoanalyst Karl Menninger and his classic *Man Against Himself *(1938) as key (the 1960s articles also reference this quite consistently). But why choose a little-known symposium in Maryland, or hospitals in New York and Pennsylvania, as key *origin* points for the *current* renderings of ‘self-harm’?

The self-evidence of ‘self-harm’ as ‘female cutting’ starts to fracture if traced back before the late 1960s. For example, Chaney notes that the behaviour of ‘self-cutting’ is ‘not emphasised in nineteenth-century writings’ (2011b: 280). In a case study of ‘self-mutilation’, published in 1932, the self-mutilating patient ‘manually fractured phalangeal articulations of the left hand. … (On the next night she dislocated both thumbs)’ (Conn, 1932: 252).

The emphatic gender dynamic observed both in the contemporary and the 1960s texts did not figure in Menninger’s work during the 1930s. Most striking is his case study of a ‘self-mutilating’ male mechanic:

His arm jerks, carefully observed, proved nearly always to be body blows … his hands were covered with the scars of minor injuries. ‘Whenever I get a knife in my hand’, he said, ‘and naturally I have to do that a lot, I always cut myself’ … Three of his teeth were missing as a result of backhand blows given himself in the mouth while working with heavy wrenches. (Menninger, 1935: 418–19)

This case is not seen as any more or less typical than the mutilations of a ‘rather pretty woman of thirty’ who killed her baby with a hammer and then amputated her own forearm by having it run over by a train (ibid.: 408–9).

A comment made in 1937 about potential roots of ‘physical self-mutilation’ named two possible mutilative behaviours that sprang to mind, but seem strange to a post-1960s audience: ‘[i]nfantilism, underdevelopment of the sexual organs, and homosexuality may be the basis of physical self-mutilation (scratching the nipples, mutilating the sex organs)’. It went on to speak of ‘[s]elf-mutilation by tickling the palate to provoke vomiting, letting blood from the nose or exposure to cold’ (Dabrowski, 1937: 6, 18). To label self-induced vomiting as ‘self-mutilation’ seems strange in the modern environment where ‘bulimia’ is produced as a separate (but associated) phenomenon. Even as late as the 1950s, a ‘unique case of self-mutilation’ was reported in 1953 where, after some months of diagnosing the problem as bronchitis, it was established that the 42-year-old woman in question had been inserting sewing needles into her chest (Mann, 1953: 220). There is no attempt to structure these examples through an assertion of the primacy of ‘female cutting’.

This is not to say that all self-mutilators reported before the 1960s could *not* be fitted into the modern phenomenon. At least one psychoanalytic case study (Emerson, 1913) exhibited several similarities to the 1960s phenomenon. However, there was no attempt at a general collapse of self-mutilation into ‘females cutting’. This is something that distinguishes late 1960s work from earlier efforts to categorize self-mutilation.

This effort was extensive and pervasive; these two structuring characteristics are related in turn here. The earlier (1967–70) studies established a ‘typical cutter’, summed up by Graff and Mallin: ‘The typical cutter was an attractive young woman, age 23, usually quite intelligent’ (1967: 36–7). Differing clinical diagnoses were irrelevant according to Goldwyn, Cahill and Grunebaum, ‘because they [the patients] were all attractive young women’ (Goldwyn, Cahill and Grunebaum, 1977: 583). Robert C. Burnham noted that ‘[a]n interesting epidemiological feature of this syndrome is that it occurs predominantly in young attractive females’ (1969: 223). Graff remarked that ‘[w]ith few exceptions, the wrist slasher is an attractive, intelligent woman’ (1967: 62). The stability of this profile was noted in accounts after 1970. Stuart Asch, working at the Mount Sinai Hospital in New York, reported ‘a new clinical picture that is almost rigidly consistent’ (1971: 603). A different group of clinicians at this hospital noted that ‘[m]any observers have been struck by the similarities in cutting gestures and the patients’ personality types’ (Rosenthal, Rinzler, Wallsch *et al.,* 1972: 1363).

Rinzler and Shapiro observed that ‘[t]he patient is almost invariably female … (males are rare)’ (1968: 485) and Novotny claimed that ‘[t]he most striking aspect of self-cutting is its higher incidence in women than in men’ (1972: 505). Graff and Mallin argued that ‘[a]ll reports agree that most such difficulties are found in girls’ (1967: 40).^8^ Ping-Nie Pao took this further, into the realm of femininity rather than just being female. He described the males that exhibited this behaviour as ‘“pretty boys” and quite effeminate’ (1969: 197) and Asch followed suit, asserting that ‘boys who cut are quite effeminate’ (1971: 612).^9^ These articles consistently establish a typical, gendered cutter around which the ‘syndrome’ is based. The precise ways in which this inequality was established are tackled in due course.

These articles’ consistent efforts to render ‘self-mutilation’ as ‘cutting’ were just as important. Simpson demonstrated an awareness of this difference, but still emphasized the importance of the particular form: ‘[s]elf-mutilation, specifically in the form of wrist-slashing, is a relatively common phenomenon’ (1975: 429).^10^ The words ‘cutting’ or ‘slashing’ (*specific forms* of ‘self-mutilation’) were consistently used in the article titles, and participants at the Chestnut Lodge symposium were just as explicit, collapsing ‘mutilation’ into ‘cutting’.

Burnham opened the symposium’s discussion (on ‘Impulsive Self-Mutilation’) by arguing that ‘the impulsive, intentional cutting of their own skin is a major symptomatic act’ (1969: 223). Podvoll saw ‘cutting as the preferred form of mutilation’ (1969: 213) and John Kafka argued for the primacy of cutting, but included another form of ‘mutilation’ alongside it, describing his patient ‘whose *foremost* symptom consisted of cutting herself and interfering with wound healing’ (1969: 207). Graff in Pennsylvania spoke of ‘the primary symptom of the slash’ (1967: 64) and Goldwyn, Cahill and Grunebaum labelled wrist-cutting ‘a prominent and preferred form of impulse discharge’ (1967: 583). This collapsing of self-mutilation into cutting, slashing, or scratching occurred throughout these ‘classic texts’, a crucial structuring characteristic. Again, this behavioural stereotype is explicitly based in the 1960s literature, and will be deconstructed below.

## Identity, authoritative knowledge and the reproduction of stereotypes

Tracing references and isolating a point at which similarities become apparent (as shown above) is all very well.^11^ However, two points need to be explored at this juncture. First, in some of the most recent publications (from approximately 2007 onwards), the explicit nature of this textual, reference-based link decreases; contributions such as Sutton (2007), Tantam and Huband (2009) and the Royal College of Psychiatrists (2010) do not include the Chestnut Lodge symposium, Grunebaum and Klerman or Graff and Mallin in their references. So we must ask how it is that these reports are still (re)producing the stereotypes (Adler and Adler do cite them, but in a cursory way [2011: 14]). Second, it is just as urgent to come to a position on how these structuring characteristics of ‘cutting’ and ‘femininity’ travel from the north-eastern United States to the UK, where many of these texts are produced. It is important to make clear that it is not being suggested here that UK clinicians are even necessarily aware of work produced more than 40 years ago in Pennsylvania, New York, or Maryland. That said, it is notable that after the Chestnut Lodge symposium was published in the *British Journal of Medical Psychology* in 1969, the first studies begin to appear in Britain: as an MPhil thesis at the Joint Maudsley and Bethlem Royal Hospital (Waldenburg, 1972), at Guy’s Hospital (Simpson, 1975), and at the London Hospital and Epsom’s Long Grove Hospital (Gardner and Gardner, 1975). However, the issue of the ‘transmission’ of this behaviour is much more pervasive and subtle than simply a North American symposium published in a British journal.

Roger Smith argues that ‘knowledge of people changes the subject matter. … When we develop our knowledge of human beings, we do not just change knowledge but potentially change what it is to be human’ and this can bring ‘into being new ways of thinking, feeling, acting and interacting’ (Smith, 2005: 56). Ian Hacking pursues a similar argument, that ‘[t]he systematic collection of data about people … has profoundly transformed what we choose to do, who we try to be and what we think of ourselves’ (1990: 3). Rhodri Hayward adds that ‘[l]abelling someone as a kleptomaniac for instance, does not simply change the way we regard their behaviour; it also changes the way they understand their own motivations and the ways that they behave. It initiates what [Hacking] has termed a “looping effect” – an ongoing process of feedback between language, practice, category and person’ (Hayward, 2011: 525; Hacking, 2002). So the 1960s studies appear less relevant as the more modern studies reproduce their findings; as self-harm becomes further entrenched as ‘female cutting’, the more people gendered as female have access to a resonant behavioural pattern said to signify ‘distress’ – the ‘looping effect’.

One result of this is that historical roots of a behavioural pattern are disguised by its success – the behaviour becomes not only self-evident, but *self-sufficient*, without the need for a history. To borrow de Certeau’s elegant phrase, the success works to ‘ceaselessly signify and conceal’ its historical, contextual roots (1986: 172). People ‘make themselves up’ (to use another of Hacking’s terms) as ‘cutters’ or ‘self-harmers’; this stereotyped identity can travel as fast, as widely and in as many diverse ways as any other piece of information. This helps to explain why the studies considered here no longer appear necessary in the Royal College of Psychiatrists’ reports, or why they are less prominent in the contemporary literature: the behaviour has ‘looped’ or become established enough to be self-sufficient and self-evident. It is no longer necessary to display prominently, invoke, or even acknowledge its source.

The existence of ‘looping’ and of ‘making up’ oneself should not be presumed, or treated like an article of faith: these processes and the *reciprocity* that they foreground in the formation of identity can be glimpsed in action in these texts. Crabtree reported that the ‘self-mutilating patient is quickly assigned the label of “slasher,” “cutter,” or “mutilator”’ (1967: 91). This seems a particularly top-down imposition, but two years later Podvoll observed a more subtle process. He claimed that ‘self-mutilating patients rapidly assume an “identity”. … They become known to both patients and staff as “cutters”, “slashers”, “slicers” and “scratchers”’ (1969: 213). This assumption of an identity – involving both staff *and *other patients – gives a flavour of the mutual process at work here, with definitional work accomplished on both sides (although accessed solely through the psychiatrist’s account). This example also shows how generic ‘mutilation’ is transformed into *more specific* kinds of behaviour, ‘cutting’ or ‘slashing’ (this narrowing is considered in more depth below).

Adler and Adler’s internet ethnography in *The Tender Cut* contains a chapter on ‘Becoming a Self-Injurer’. Their key distinction is between people who started to injure themselves prior to 1996, who primarily reported experiencing themselves as ‘self-inventing’ the behaviour (2011: 56–7), and those who began after that date, who recall hearing about the behaviour first (either through the media or friends) and wanting to ‘try it’ (ibid.: 57–60). This shows an important awareness of the ways in which stereotypes might achieve conscious prominence at a certain point, and become widely available for ‘making up’ oneself.

This assumption/imposition of an identity, and the narrowing/stabilizing of a stereotype can usefully be considered as two aspects of the same process of ‘looping’ or ‘feedback’. Ideas about ‘human identity’ – partially because many humans experience themselves as ‘self-conscious’ – are curiously potent. Once these stereotypes become established, it grows increasingly difficult to see how the ‘professionally produced’ diagnoses can be considered as meaningfully distinct from behaviours performed by those constituted as subjects of these analyses: in short, the already unstable boundary between ‘authoritative knowledge’ and ‘subject’ collapses.

To approach ‘self-harm’ in this way means that the ways in which people produce, relate to and reproduce authoritative knowledges about ‘self-harm’ can become the object of analysis; the behaviour is not simply seen as inevitable or transhistorical, either as a basic response to some sort of undifferentiated ‘distress’ (Royal College of Psychiatrists, 2010: 21) or, more flamboyantly, as ‘a behaviour that is culturally and psychologically embedded in the profound, elemental experiences of healing, religion and social amity’ (Favazza and Conterio, 1988: 27).

## 1960s intellectual work on gender and behaviour: ‘Femininity’, ‘cutting’ and ‘slashing’

Having established that contemporary ‘self-harm’ concerns are substantially rooted in the 1960s, and that the possible identities forged there are potent resources for continuing human action and (self-)understanding, it is vital to subject these identities to historical critique (following Scott, 2007). It is not enough simply to *situate* these stereotypes in history, they must be deconstructed and shown to be *contingent*.

The success of this identification of ‘self-harm’ with ‘female cutting’ should not disguise that it was achieved through practices of exclusion and emphasis. Structural characteristics that underwrite ‘stereotypes’ or ‘identities’ do not just appear out of thin air, or out of the monolithic, important-sounding substitutes of ‘culture’ or ‘environment’ or the ‘experience’ of clinicians. Brickman offers the astute argument that self-harm is stereotyped as female because:

… the medical discourse on ‘delicate’ cutting pathologises the female body, relying on the notion of femininity as a disease … one begins to wonder if ‘mutilation’ would be used so readily to describe wounded skin on a less appealing body. (Brickman, 2005: 89, 98)

There is clearly a productive line of analysis, but when accounting for this situation historically, Brickman attributes it to a rather indistinct ‘particular social bias’ (2005: 88). This may indeed be the case – with a net cast that wide, how could it not be? Pushing further, there are far more prosaic, practical ways in which this phenomenon was produced as feminine.

Adjusting the composition of the groups under consideration was quite common and perhaps the most obvious exclusionary process. Graff and Mallin’s sample was originally ‘21 females and one male’. However, ‘[t]he male, a 56-year-old dentist, was excluded from the study because we felt he was atypical’ (1967: 36). In another study, 11 men (out of 35 patients in total) had a history of wrist cutting, ‘but the findings were so different from those of the women that they will be presented in a separate paper’ (Rosenthal, Rinzler, Wallsch *et al.,* 1972: 1363). This paper was never published as far as I am aware, so the presentation of a syndrome that affected only women was artificial to say the least. A 1972 study in London comprised 24 patients who had mutilated themselves, 5 males and 19 females. Of these, 2 males and 13 females were repeat self-cutters, but ‘in view of the extremely small number of men there seemed little point in comparing them formally with matched subjects’. Thus the men were excluded from the crux of the project (Waldenburg, 1972: 16).

Ping-Nie Pao’s gendered exclusions were perhaps the most audacious, marrying numerical exclusion with basic gender stereotype: ‘Of the 32 patients in our series, five were coarse cutters and 27 were delicate cutters’. Unsurprisingly, women predominated among ‘delicate cutters’ and men among ‘coarse’: ‘[o]f the five coarse cutters, four were male and one female, whereas 23 of the 27 delicate cutters were female’ (Pao, 1969: 195). While these groupings were artificial and obviously gendered, it is of interest to note that the divides were not completely ‘clean’ in either case. However, the four male ‘delicate cutters’ were not mentioned again, except, as seen earlier, to call them ‘“pretty boys” and quite effeminate’ (ibid.: 197). In other cases, authors selected patients themselves from clinical records (e.g. Asch, 1971). Thus the exclusion of men was achieved by the assumption that females are exemplary.

In these specific articles from the 1960s and 1970s there are several ways in which samples were produced as gendered. This is even before it is recognized that these studies were predominantly from private psychiatric inpatient facilities where women outnumbered men in this period. However, further analysis of these institutions’ characteristics is beyond the scope of this article.

The work around ‘cutting’ is, if anything, even more strenuous. Edward Podvoll and Harold Graff (from Chestnut Lodge and the Eastern Pennsylvania Psychiatric Institute respectively) both made a conscious effort to promote ‘cutting’ or ‘slashing’ even while noting many other symptoms. Graff claimed that ‘slashing is actually a part of a whole constellation of symptoms which make up a major behavioural pattern … not only the primary symptom of the slash, but the sexual promiscuity, the alcoholism, the addiction to drugs’ (1967: 64). ‘Slashing’ was explicitly cast as ‘primary’, and the other behaviours in this pattern were relegated to secondary status without explanation. He acknowledges a ‘whole constellation of symptoms’, but this does not get in the way of the ‘wrist-slasher’ of the title, or problematize the syndrome pattern he was establishing by their exclusion.

Podvoll speculated even further about the primacy of ‘cutting’, observing that although ‘the self-destructive potential of these patients often extends to burning, biting, toxic ingestion and starvation, they usually return to cutting as the preferred form of mutilation’. This was supposedly because ‘it seems to be the most difficult for the staff to manage – as though no other outward signs would more adequately express their inner distress’ (Podvoll, 1969: 213). Podvoll shows that there are many other symptoms that, while not excluded, were certainly not emphasized, contributing to the establishment of the behavioural stereotype.^12^

In two of the articles from the Mount Sinai Hospital (1971 and 1972), a very distinctive form of mutilation was mentioned, that of carving letters or words into the skin. The latter article started with a list, in a similar manner to Podvoll and Graff: ‘Almost half engaged in some other form of self-mutilation, including burning themselves with a cigarette or an iron; scratching, gouging or rubbing glass fragments into their faces; repeatedly traumatising fresh fractures; and carving initials in their skin’ (Rosenthal, Rinzler, Wallsch *et al.,* 1972: 1364). This last behaviour was also mentioned the previous year by Asch (who was using the same data as Rosenthal *et al.*):

… [t]he self-mutilation performed by one girl … [involved the use of] a knife to scratch the letters ‘LOVE’ on her thigh, just deep enough for blood to flow. … When I [Asch] wondered at the choice of the letters ‘LOVE’ she confessed that her original impulse was to cut ‘HATE’ but that she had stopped herself ‘because that didn’t seem very nice’. (Asch, 1971: 612–13)

This cutting of letters was not seen as going against or modifying any kind of syndrome pattern or stereotype, even though this kind of communication in words or letters seems at least as significant for the patient as the cutting.

Kafka and Novotny recorded additional distinctive behaviours. Kafka’s patient’s foremost symptom was cutting herself and interfering with wound healing, but she ‘also swallowed pills indiscriminately, refused to take medication, and/or cheated on taking medication’ (1969: 207). Thus out of five ‘deviant behaviours’, three involved medication, and only one involved cutting. One may also assume that refusal to take medication and ‘cheating’ on taking it would not have been classed as ‘mutilation’, but this was not seen to affect her status as a ‘mutilator’ (‘swallowing pills indiscriminately’ is rather more ambiguous^13^). In a similar way, Novotny described ‘patients who insert in their lacerations needles and similar foreign bodies’, but again, this action is not seen as nearly as important as the act of self-cutting, which was constitutive of the stereotype and his article’s title, ‘Self-cutting’ (Novotny, 1972: 510).

Crabtree’s patient’s symptoms were so varied that they were not even unified by the attribute of actions involving her own body. When she was admitted to an adolescent unit,

… [f]acial scratching persisted, soon replaced by deep lacerations of the forearms … her inability to refuse a dare. She attempted to break windows; she set fires at various places … she took an overdose of aspirin requiring emergency treatment; she swallowed a needle. (Crabtree Jr, 1967: 93)

This is a very broad range of ‘deviant’ or ‘pathological’ behaviours. Why these patients, as Crabtree noted above, were labelled ‘slashers’ is not immediately obvious.^14^

Rhetorical formulations by Simpson and Pao conceived non-cutting ‘mutilations’ as ‘other symptoms’, somewhat indeterminate, but definitely secondary. Simpson claimed that ‘*[o]ther varieties* of self-mutilation may occur in the same patients [i.e. ‘cutters/slashers’], especially burning themselves with cigarettes, dermatitis artefacta and tattooing’ (1976: 295; emphasis added). Similarly, Pao argued in a startling list, that

… [a]s a rule, in *addition* to the delicate self-cutting, each *cutter* had *other symptoms* which [included] eating problems (bulimia and anorexia), mild swings of depression and elation, brief moments of lapse of consciousness … absconding from the hospital, promiscuity, suicidal ruminations, ingestion of sharp objects or intoxicants, breaking window-panes or furniture, burning themselves with lighted cigarettes, attempted arson, and so on. (Pao, 1969: 196; emphases added)

The rhetorical strategy of designating non-cutting behaviours as ‘other’ or ‘additional’ helped to establish the primacy of cutting and to actively constitute one of the key structural characteristics that persists in the modern renderings of ‘self-harm’.

As well as the articles that explicitly promoted ‘cutting’ as somehow primary among self-mutilative behaviours, there were many instances of slippage. Asch described a patient with very varied ‘self-destructive’ desires who ‘felt that she must do something, squeeze something, to dig at her wrists with her fingernails, to smash something, to cut herself’. Even with these varied desires, her behaviour was only ever referred to as ‘cutting incidents’ (Asch, 1971: 611). (This may have been because the only desire she ever *acted upon* was cutting, but this is not explained or even mentioned again.) Burnham reported that ‘My patient was able to describe the subjective experience of her cutting from time to time. She was an articulate woman of very superior intelligence and extensive symptomatology who cut, burned, hit, abraded and scalded herself’ (Burnham, 1969: 225). Yet again, the ‘extensive symptomatology’ was rendered as ‘her cutting’ without explanation. This intellectual work actively homogenized varied clinical symptoms into ‘cutting’, producing one of the pillars of modern ‘self-harm’.

## Why cutting? Pain, blood, psychodynamics and suicide gestures

Having established that ‘cutting’ was actively promoted, emphasized and highlighted from the ‘multifarious’ observed behaviours of these patients, it is important to attempt an explanation. There are three possible and interrelated reasons for this. First, is that ‘cutting’ was supposed to be a painful behaviour, and yet was not experienced as such by many patients (a concern that is less immediately relevant to the act of overdosing, for example). ‘Cutting’ was also an act that produced blood in significant quantities (unlike either ‘overdosing’ or self-burning). Both these characteristics made ‘cutting’ appear particularly interesting to psychodynamic or psychoanalytical clinicians. Finally, the ‘self-cutting’ literature grows out of a concern to differentiate these actions from attempts at suicide.

The 1960s articles were influenced by and structured through psychoanalytic assumptions, although this was rarely mentioned explicitly. Simpson did state that most of the studies in his survey operated ‘on the basis of some pre-existing psychodynamic interpretation’ (1975: 429), and, in the secondary literature, Favazza and Conterio state that ‘[m]ost of the detailed information about chronic self-mutilators has come from a handful of psychoanalytically oriented case studies’ (1989: 23). However, neither mentions that this intellectual context has specific implications for the form of the clinical object. Nonetheless, specific psychoanalytically influenced concepts contribute substantially to emphases on an abnormal lack of pain and the presence of blood and they bring the action of ‘cutting’ to prominence.

The absence of pain when cutting was seen as one of the strongest unifying features of the early syndrome. Simpson claimed that the ‘typical absence of pain during the actual cutting is a very common and intriguing feature’ (1976: 298). Others note that ‘[c]haracteristically, they did not experience pain but felt relief as the flowing blood “drained something bad from them”’ (Goldwyn, Cahill and Grunebaum, 1967: 584). Grunebaum and Klerman asserted that the ‘absence of pain during the actual cutting is an interesting feature observed in many patients’ (1967: 529). Graff and Mallin noted that ‘the cutter is able to slash herself without pain or with pleasure’ (1967: 40). Even when patients did feel pain, they were cast as atypical: ‘Unlike most patients of this sort, [this patient] was not amnesic or anaesthetic for these episodes in general, but cut and burned (with cigarettes) herself with conscious gratification in the pain she was inflicting and experiencing’ (Burnham, 1969: 225–6).

This lack of pain is partially brought to prominence in this psychoanalytic context by Freud’s ‘pleasure principle’ which holds that, subconsciously, humans always seek to experience pleasure and avoid pain and so problematizes actions of self-mutilation. An early statistical (rather than clinical) study on ‘self-mutilation’ from a large psychiatric hospital in New York, explicitly referenced Freud, noting an ‘apparent violation of the “pleasure principle” through self mutilation’ (Phillips and Alkan, 1961: 428). They presented some of the ‘various theories [that] have been formulated to explain this paradoxical type of [behaviour]’, rehearsing Menninger’s postulations on ‘focal suicide’ and Otto Fenichel’s discussion of religiously inspired self-castrations. These discussions focus upon how these theories remove the contradiction between subconsciously motivated ‘self-mutilation’ and the ‘pleasure principle’ that supposedly rules the unconscious (ibid.: 428–9). Although these theories were not dealing with the mutilations typically associated with the DWCS (self-castration, for example), they show that prominent analysts (Menninger and Fenichel) had *attempted to square supposedly painful, ‘mutilating’ acts with Freud’s pleasure principle*.

As this ‘painless cutting’ did not contravene Freud’s pleasure principle, it could be more easily slotted into a psychodynamic schema. The clinicians did not have to explain away the pain of self-mutilation with some form of more complicated ‘exchange mechanism’ such as Menninger’s (who *excused* the pain and loss of self-mutilation by claiming that it was averting the greater loss of suicide, trading pain and perhaps an appendage in return for viable psychic existence^15^). Therefore mutilative behaviours that were accompanied by a lack of pain were apt to be more interesting to these clinicians. As noted, Simpson called it a ‘common and *intriguing *feature’ (1976: 298; emphasis added) and Grunebaum and Klerman noted that it was ‘an *interesting *feature observed in many patients’ (1967: 529; emphasis added). The psychoanalytic context further emphasized the lack of pain for Graff and Mallin, who claimed that the ‘pleasure experienced in the act of slashing is most striking’ (1967: 40). Thus the presence of a predominantly psychodynamic context helped to emphasize the pathological anaesthesia, which might account for the promotion of cutting over many of the actions listed above, such as ‘overdosing’, ‘breaking windows’, or ‘eating problems’.

The second explanation for the prominence of ‘cutting’ involves the unique place of blood in the clinical object of the 1960s and 1970s. Asch simply stated that ‘it is the presence of blood that is important’ (1971: 612), and other psychiatrists claimed that the *patients* ‘spoke of being “happy” at the blood, happy to *see* it, being “fascinated” by it’ (Rosenthal *et al.,* 1972: 1366–7). Another article from the same hospital but with an earlier patient sample, noted that for one particular patient, ‘the sight of her own blood often prevented a retreat into a remote and terrifying world of fantasy’ (Rinzler and Shapiro, 1968: 487).

Simpson’s literature survey concluded that ‘[b]lood has a special significance for the self-mutilator’ (1976: 298) and reproduced this startling personal communication by a doctor relating one patient’s reaction to blood:

The real thing that excites me most is to see my blood. … Deep rich red, the colour, the velvety warmness … invaded by liquid rubies or a vintage claret – it moves slow like the birth of a child or like wearing an Afghan coat on a cold day. (Simpson, 1976: 299, 314)

These striking reactions to blood were not uncommon. Asch also noted a ‘specific visual phenomenon. … One patient … explained, “There was too much white, white nurses, white doctors, white sheets, white walls. It was such a relief to cut and see the red blood appear”’ (Asch, 1971: 612). These testimonies show how the emphasis on blood was, at least partially, patient-led.

While a focus upon blood was not a psychoanalytic staple to the same extent as penis envy or castration concerns, it was analysed and mediated in symbolic ways that were psychoanalytically inflected. It is important to ask *what it was* about blood that was seen as important enough for the clinicians to include it (and patient testimony about it) in the texts. Simply arguing that it was deemed important because the patients thought it so is insufficient: patient testimony only makes it into print if the clinicians put it there.

Some blood concerns were explicitly mediated by observing clinicians. Asch uses Menninger’s concept of ‘focal suicide’ with explicit regard to blood: ‘blood-letting could be a concrete manifestation of one of the classical dynamics of depression, a little suicide’ (1971: 615). Kafka mediated his patient’s testimony with a concept borrowed from prominent psychoanalyst Donald Winnicott: ‘Blood was described by the patient as a transitional object. In a sense, as long as one has blood, one carried within oneself this potential security blanket’ (1969: 209). Thus, the emphasis by some patients on the heightened status of blood attained further significance through psychodynamic explanatory devices, cementing its position in the syndrome, at the expense of ‘mutilative’ behaviours such as self-burning that do not produce comparable amounts of blood. There are infrequent attempts, too, to relate the production of blood to vicarious menstruation (Siomopoulos, 1974; Gardner, 2001) which also feeds into the feminization of the syndrome. There are also other practical, but little-mentioned, ideas, such as Podvoll’s contention that ‘self-mutilation’ was most difficult for staff to manage, which explicitly links the behaviour to the context inside a psychiatric hospital.

Finally, the act of cutting is prominent because the early articles spent much time differentiating the actions of their patients from attempts to kill themselves. Goldwyn, Cahill and Grunebaum were explicit: ‘these individuals lack obvious suicidal intent’ and their ‘self-inflicted injury to the wrist’ is ‘not an attempt at suicide’ (1967: 583, 587). Novotny’s opening sentence also differentiated self-mutilation from suicide: ‘Minor self-inflicted cuts of areas of the skin constitute a symptom carried out without serious suicidal intent’ (1972: 505).

This was a conscious attempt to differentiate between *outwardly similar* actions or behaviours. Graff and Mallin noted that ‘[m]any hospitals are experiencing an influx of patients who have made several suicide attempts by wrist-slashing’. Though this seems misleading, the emphasis of this sentence was that hospital staff *incorrectly* assumed themselves to be experiencing this surge in ‘attempted suicide’. This is made obvious two paragraphs later by the observation that ‘[s]elf-mutilation by wrist cutting has been submerged in the statistics of suicide and attempted suicide’ (1967: 36). These authors were focusing on a behaviour pattern that resembled a suicide attempt closely enough for medical professionals to ‘misinterpret’ it (see also Grunebaum and Klerman, 1967: 532; Rinzler and Shapiro, 1968: 487; Pao, 1969: 195; Asch, 1971: 612; Simpson, 1976: 286).

A 1950s article comparing ‘suicide’ with ‘attempted suicide’ noted more generally that ‘cutting’ had the lowest completion rate for all methods of attempted suicide (Schmid and Van Arsdol, 1955: 282). Nelson and Grunebaum, talking of apparently genuine ‘suicide attempts’, observed that wrist slashing is ‘a notoriously poor method of suicide’ (1971: 1348). Thus, ‘cutting’ is seen as a behaviour with enough *symbolism* to be considered ‘suicidal’ yet with low enough ‘lethality’ to be thought problematic, either as a ‘gesture’ or as aiming at something else entirely. *Thus, cutting is right at the source of the confusion that these texts are trying to address.*

This may seem confusing as the ‘wrist’ part of the archetype does not feature in contemporary literature, and it has become self-evident that ‘self-harmers’ are not attempting to kill themselves. However, the modern phenomenon of ‘self-harm’ is rooted in texts that were concerned with this differentiation and confusion; other actions (such as interfering with wound healing, self-burning, or arson, all mentioned above) would not generate the confusion that these articles were trying to clear up. Thus ‘cutting’ becomes more noticeable. It might speculatively be argued that ‘self-poisoning’, which is *also* considered of low ‘lethality’ and has symbolic resonance with attempts to kill oneself, does not emerge so resonantly in the USA due to the psychodynamic context (emphasizing blood and pain); this method is, however, hugely significant in the UK, where psychoanalysis is less popular and established (see Kessel, 1965).

The specific intellectual context of these studies helps to explain the emergence of this syndrome, through their potential to single out or make interesting *one *of the ‘multifarious’ behaviours. Psychoanalytic terminologies and explanatory frameworks emphasized those symptoms with the most analytic potential – those involving blood and questions of pain – and thus threw cutting into sharper relief. The articles also sought to address confusion around ‘suicide attempts’, which elevated ‘mutilations’ that might be ‘confused’ with suicide attempts.

## Conclusion

Conceptual literature on the historical nature of disease categories and psychiatric diagnoses throws new light on the history of ‘self-harm’. Durable assumptions and emphases (structural characteristics) concerning the subject profile and archetypal behaviour exist within psychiatric articles that attempted to establish this syndrome. Certain behavioural assumptions and gendered exclusions structure and produce the profile; these exclusions and emphases still largely produce the modern literature. These structural characteristics filtered a range of subjects and behaviours, producing a homogenous illness pattern involving a central act of cutting and a patient gendered female.

Once established, produced through specific exclusionary practices, a behaviour pattern can become self-sufficient, transcending its formative contexts; a wide variety of people can ‘make themselves up’ in relation to it. It is no longer simply American young women in psychiatric inpatient facilities who are seen as at ‘high risk’ for ‘self-harm’. Now, people identified as South Asian women, or lesbian, gay or bisexual, are becoming increasing prominent groups at ‘high risk’ of ‘self-harm’ (e.g. King, Semlyen, Tai *et al.,* 2008; Husain, Waheed and Husain, 2006). Those identifying with or identified within these stereotyped groups are increasingly able to reproduce, refashion and perform them. Regarding ‘transmission’, it is interesting to note that with only a few exceptions (for example, Waldenburg, 1972; Gardner and Gardner, 1975) concerns in 1960s and 1970s Britain about harmful ‘distress behaviour’ of ‘young women’ centred around a supposed epidemic of ‘self-poisoning’ or ‘overdosing’ which was also called ‘parasuicide’ (e.g. Kessel, 1965; Kreitman, 1977). It was not until the mid-1980s at the earliest that ‘self-cutting’ became prominent. This does not mean that ‘self-cutting’ was ‘not happening’, in the UK, simply that the behaviours likely to come to light, to come into the orbit of clinicians (and stand out) in US psychiatric inpatient facilities, and those brought to National Health Service hospitals, were substantially different.

When stereotypes become established (which might register as an ‘increase’, or when hospitals become aware of ‘self-cutting’ as a problem), the profiles and archetypes laboriously constructed in the psychological literature become reinforced as people ‘make themselves up’ in relation to them; these people, in turn, become the basis for further commentary and dissemination of the stereotype in a complex and circular process. Time and space, context and practice, are crucial to the emergence and maintenance of any psychiatric or medical object. These modern experts and (perhaps less surprisingly) media commentators remain unaware of the very distinctive exclusionary practices that divided by gender and structured behaviours so that only certain characteristics ‘made the cut’ into self-mutilation stereotypes.

But in the end, to quote Roger Cooter, ‘So what? Why bother?’ (Cooter, 2010). For one thing, analysis of the *source* of the gendered exclusions that produce ‘self-harm’ can serve as a counter to a certain type of antifeminist assertion. For example, in the ‘World of Pain’ documentary, it is remarked that 1970s feminism – characterized as ‘women having it all’ – is ‘paid for’ with the increased incidence of ‘self-harm’ in women (Syal, 2009). The practices that gender self-harm must *not* be allowed to undercut further any gains made by second-wave feminism. The kind of analysis pursued throughout this article critiques and exposes the active sexism present in a historically ‘feminine’ pathology. In a more straightforward (but no less important) way, the analysis may also help clinical outreach by ‘self-harm experts’ to those identifying as male.

However, there is a broader applicability for analyses of this kind. The central statement of this article and its theoretical underpinnings might be summarized thus: behavioural patterns, diagnoses and illness categories are made by human beings, in specific contexts through specific intellectual and practical processes of sense-making. Behaviours/diagnoses such as ‘self-harm’ are *not* some kind of surface expression of monolithic deep causes such as ‘distress’ or to be collapsed into the esoteric languages of genetics, biology, or neurochemistry (e.g. Brent, 2011) – or the now unfashionable ego, Oedipus complex, or ‘penis envy’.

This analysis aims to practise ‘history-writing as critique’, characterized by Scott as ‘the attempt to make visible the premises upon which the organising categories of our identities … are based, and to give them a history, so placing them in time and subject to review’ (2007: 34–5). The premises of ‘self-harm’ – specifically ‘female cutting’ – have been exposed as contingent, rooted in specific concerns and practices in the 1960s in North America. Contemporary ‘self-harm’ is not a natural, eternal, or transcendental object; it is thus subject to review and the possibility of change. Thus, on a more intimate level – necessarily a more speculative, but no less important, one – if we begin to understand psychological categories *critically*, as one small part of potentially infinite* methods of sense-making*, the number of people who feel themselves to be trapped inside diagnoses, victims of timeless behavioural patterns or dictated to by gender-specific coping strategies may decrease. It is also just possible that an awareness of the contingency and artificiality of the stereotypes around ‘self-harm’ might contextualize and undercut the feeling of an unwanted compulsion to perform it.

## Notes

Thanks to Mark Jenner, for copious feedback on the MA dissertation upon which this article is based; to Rhodri Hayward, Åsa Jansson and Daniel DeGroff for comments on this version of the research; and for comments by anonymous reviewers for *History of the Human Sciences*.

1. The *Independent* led with Frith’s (2004) ‘New Figures Reveal Hidden Epidemic of Self-Harm’ which carried criticism of accident and emergency department staff. The *Daily Telegraph* and *Guardian* responded with less than charitable assessments (of the ‘self-harmers’) from McKinstry (2004) and James (2004). For a discussion, see Sutton (2007: 116–23).

2. Six characteristics that run through the phenomenon of ‘self-harm’ are identified in Millard (2007): femininity, cutting, pathologized eating and/or vomiting practices, the symbolism of blood, lack of pain during cutting and the therapeutic value of cutting for the patient. There is no space to tackle them all in this article.

3. Wilson, by his own admission, has no space to do this. He makes ‘no attempt to *explain *the various shifts of meaning which will emerge’ calling his article ‘strictly internalist’ and ‘textual’ (Wilson, 2000: 283; emphasis added).

4. However, a gendered stance re-emerges through the use of feminine pronouns: ‘[f]ew behaviours are as shocking as a young person intentionally carving herself bloody’ (Plante, 2007: 38).

5. They reference Ross and Heath’s ‘A Study of the Frequency of Self-Mutilation in a Community Sample of Adolescents’ as contesting the feminization of ‘self-injury’ from 2002 (Tantam and Huband, 2009: 4).

6. Simpson’s article is a convenient ‘cut-off’ point because it is the first literature review (rather than clinical study) and is very confident about the existence of a syndrome: ‘there exists a clearly identifiable condition of self-mutilation, usually involving wrist-cutting, which exhibits much of the stability of a syndrome’ (1976: 310). Thus the stereotype had been established by 1976.

7. She also traces a line from Menninger (1938) to Favazza (1996) through Grunebaum and Klerman (1967) (Plante, 2007: 7–8).

8. Graff repeats this sentiment in his own article: ‘Most studies have shown that the majority of them [cutters] are women’ (Graff, 1967: 62).

 9. This ‘feminizing’ linkage is also noted in Simpson (1976: 292).

10. He shows awareness of this distinction elsewhere, claiming that the ‘commonest form [of self-mutilation is] wrist cutting’ (Simpson, 1976: 287). The emphasis here on ‘*wrist* slashing’ is interesting with regard to questions of ‘suicidal intent’. See Millard (2007).

11. The idea of an – admittedly messy and uneven – ‘progression’ towards modern ‘self-harm’ through some works by Tom Main (1957) at the Cassell Hospital and Stanton and Schwarz (1954) at Chestnut Lodge (among others) is explored in Millard (2007).

12. The importance ascribed to institutional influences in the motivations for psychiatrically pathologized behaviour fluctuates. ‘Self-cutting’ first is cast as an attempt by patients to affect the staff’s ability to manage them (seemingly following Stanton and Schwartz, 1954). This is followed by a retreat into a basic ‘inner distress’ that underpins the ‘self-cutting’. There is no space to interrogate this shift any further.

13. In a different take on the self-injury/self-poisoning debate, a group of Canadian ‘suicidologists’ included ‘any case of self-inflicted overdosage, asphyxiation or injury’ in their studies on ‘self-injury’ (Johnson, Ferrence and Whitehead, 1973: 101).

14. This is approached below. For more explanation, see Millard (2007).

15. He argued that ‘self-mutilation is actually a compromise formulation to avert total annihilation, that is to say suicide’ (Menninger, 1935: 466).
